# Prevalence of suboptimal drug treatment in patients with and without multidose drug dispensing—a cross-sectional study

**DOI:** 10.1007/s00228-014-1683-0

**Published:** 2014-05-08

**Authors:** Björn Belfrage, Anders Koldestam, Christina Sjöberg, Susanna M. Wallerstedt

**Affiliations:** 1 Närhälsan Dals-Ed Health Center, 668 30 Dals-Ed, Sweden; 2 Department of Geriatrics, Sahlgrenska University Hospital/Mölndal, 431 80 Mölndal, Sweden; 3 Department of Clinical Pharmacology, Sahlgrenska University Hospital, 413 45 Göteborg, Sweden

**Keywords:** Drug therapy, Health care quality assessment, Multidose drug dispensing, Overtreatment, Undertreatment

## Abstract

**Purpose:**

The aim of this study was to compare the prevalence of suboptimal drug treatment in older patients with and without multidose drug dispensing (MDD).

**Methods:**

In 200 hip fracture patients (≥65 years of age), originally recruited to a randomized controlled study in Sahlgrenska University Hospital in 2009, quality of drug treatment at study entry was compared between patients with and without MDD. Two specialist physicians independently assessed and then agreed on the quality of the drug treatment of each patient. Suboptimal drug treatment was defined as ≥1 STOPP (Screening Tool of Older Persons’ potentially inappropriate Prescriptions) or ≥1 START (Screening Tool to Alert to Right Treatment) outcome assessed as clinically relevant after individual considerations had been made, i.e. over- or undertreatment (≥1 inappropriate and ≥1 missing drug, respectively).

**Results:**

Patients with MDD (*n* = 100) differed from patients without MDD (*n* = 100) in several ways, for example by being older (87.6 vs. 81.5 years) and using more drugs (8.4 vs. 5.9 drugs). The total number (±standard deviation) of inappropriate and/or missing drugs per person was greater in MDD patients compared with patients without MDD (1.92 ± 1.52 vs. 1.06 ± 1.29, *P* < 0.0001); MDD patients had an additional 0.77 inappropriate drugs and an additional 0.09 missing drugs per person. The prevalence of suboptimal drug treatment was greater in patients with MDD than in those without MDD (86 vs. 55 %, *P* < 0.0001). Logistic regression revealed that suboptimal drug treatment was 8.0 times as common in MDD patients, after adjustments for age, sex, number of drugs, cognition, and residence (95 % confidence interval 2.4; 26.9). Corresponding figures for over- and undertreatment were 2.9 (1.1; 7.4) and 1.8 (0.8; 4.3), respectively.

**Conclusions:**

Suboptimal drug treatment, including over- and undertreatment, is more common in MDD patients than in patients who receive their drugs via ordinary prescriptions. The findings confirm safety concerns regarding quality of drug treatment in MDD patients.

## Introduction

Dose dispensing systems are widespread over the world [1]. However, systematic reviews have shown that scientific evidence is scarce [2, 3]. In fact, evidence on beneficial effects, such as improved adherence, is inconsistent [4, 5]. On the contrary, recent research has indicated safety concerns regarding the prescribing of drugs to patients within such systems. For example, patients with multidose drug dispensing (MDD) have more drugs in the medication list and the drug treatment is more often potentially harmful according to general indicators of prescribing quality [6]. Further, there is evidence suggesting that drug treatment is more seldom reconsidered in patients with MDD [7]. Indeed, a longitudinal analysis even indicates a causal relationship, that is, after transition to an MDD system a distinct and maintained change in drug treatment occurs [8].

If there is a causal relationship between MDD and suboptimal drug treatment, this is worth attention; the prescriber rather than the nursing and pharmacy services accounts for the majority of severe medication errors [9]. The need for scientific evidence is further emphasized by the fact that MDD is often used by frail patients who may have a diminished capacity to speak for themselves, i.e. older people and people who have difficulties in handling their drugs due to impaired physical or cognitive function. Moreover, MDD is used by many patients. In Sweden, for example, 8 % of people aged ≥65 years use such a system [1].

Available evidence does not allow firm conclusions concerning quality of drug treatment in patients with MDD. Indeed, to assess quality of prescribing is a delicate matter. In fact, an extensive medication list and potentially harmful drug treatment according to general indicators of prescribing quality do not necessarily imply suboptimal drug treatment. For example, a large number of drugs may be appropriate at the individual level as state-of-the-art guidelines for many conditions, including, e.g. cardiovascular disease, imply concurrent treatment with several drugs. Further, the ability of general indicators to distinguish between appropriate and inappropriate drug treatment at the individual level has not been established [10].

An approach to determine quality of drug treatment may be to use screening tools to identify potential quality problems and then to confirm or reject these by specialist physician assessments based on individual considerations for the specific patient. Indeed, when it comes to characterizing the quality of drug treatment, a medical assessment at the individual level is the key step. As for the screening procedure, the validated screening Tool of Older Persons’ potentially inappropriate Prescriptions (STOPP) and the Screening Tool to Alert to Right Treatment (START) may be useful to identify potentially inappropriate and missing drugs, respectively [11].

The aim of this study was to compare the prevalence of suboptimal drug treatment in older patients with and without MDD, that is, over- and undertreatment after individual considerations have been made.

## Methods

The study complies with the Declaration of Helsinki, and ethics approval was obtained from the Regional Ethical Review Board in Gothenburg (Dnr 095-09).

In 200 hip fracture patients (≥65 years of age), consecutively recruited to a randomized controlled study in the departments of orthopedics, geriatrics, and medicine at Sahlgrenska University Hospital in 2009 [12], quality of drug treatment at study entry (admission to hospital) was compared between patients with MDD and patients without MDD (control patients).

One general practitioner (BB) and one geriatrician (AK) independently assessed the quality of drug treatment in two steps. First, they identified potentially suboptimal drug treatment by the use of STOPP and START. These screening tools provide 65 criteria for potentially inappropriate drugs and 22 criteria for potentially missing drugs, respectively [11]. Then the clinical relevance of identified STOPP and/or START outcomes was assessed at the individual level. Thus, STOPP outcomes were assessed as not clinically relevant, i.e. not representing an inappropriate drug, if the expected benefit of the medication was judged to outweigh the potential harm, such as neuroleptic treatment in a patient with schizophrenia. Correspondingly, START outcomes were assessed as not clinically relevant, i.e., not representing a missing drug, if there was a clinical reason, such as an adverse drug reaction or a contraindication, not to treat the patient with the drug. In a final consensus discussion, the two specialist physicians reached agreement on all STOPP/START outcomes, and the clinical relevance of these. In order to keep a conservative approach to categorizing drugs as inappropriate or missing, we chose to categorize STOPP and START outcomes not possible to assess concerning clinical relevance as not clinically relevant.

Suboptimal drug treatment was defined as ≥1 STOPP or ≥1 START outcome assessed as clinically relevant after individual considerations had been made. Correspondingly, overtreatment was defined as ≥1 clinically relevant STOPP outcome (≥1 inappropriate drug) and undertreatment as ≥1 clinically relevant START outcome (≥1 missing drug). If no clinically relevant STOPP or START outcomes were present, the drug treatment was considered appropriate.

All drug treatment assessments were based on information in the medical records, including laboratory data, from hospital and primary care. These records were available due to the design of the original randomized controlled study. Further, baseline data from the original study were used, including information on risk of falls, cognition, residence, and kidney function [12]. The latter, estimated with the Cockcroft-Gault equation, was dichotomized as either ≥50 or <50 ml/min to fit the STOPP and START criteria.

### Statistics

The statistical analyses were performed using SPSS 17.0 (SPSS, Chicago, IL, USA). The Mann-Whitney and the chi-square tests were used for comparisons between MDD and control patients. Kappa statistics was used to assess inter-rater agreement. Logistic regression was performed to evaluate the association between MDD and suboptimal drug treatment as well as over- and undertreatment. The results were adjusted for age, sex, number of drugs (as a proxy for burden of disease), cognition (defined as impaired including dementia or not), and residence (defined as nursing home or not). With 100 patients in each group and an expected prevalence of suboptimal drug treatment of 60 % in control patients, the study had a power of >80 % to detect that the corresponding prevalence in MDD patients was 33 % greater.

## Results

Characteristics of the patients are presented in Table 1. Compared with control patients (*n* = 100), MDD patients (*n* = 100) were older and treated with more drugs. Further, their cognition and kidney function were more often impaired. They also lived in nursing homes to a greater extent.

**Table 1:** Characteristics of MDD and control patients. Values are given as counts (which correspond to percentages) or mean ± standard deviation

	MDD, *n* = 100	Control, *n* = 100	*P* value
Age, years	87.6 ± 5.4	81.5 ± 7.2	<0.0001
Female sex	68	65	0.65
Impaired cognition, including dementia	74	16	<0.0001
Nursing home resident	55	5	<0.0001
eGFR <50 ml/min	59	33	0.0002
Number of drugs	8.4 ± 3.6	5.9 ± 3.8	<0.0001

*eGFR* estimated glomerular filtration rate, *MDD* multidose drug dispensing

The prevalence of STOPP/START outcomes as well as suboptimal drug treatment is presented in Table 2. The kappa value of the inter-rater agreement for STOPP and START outcomes was 0.52.

**Table 2:** Prevalence of STOPP/START outcomes as well as suboptimal drug treatment in MDD (*n* = 100) and control (*n* = 100) patients. In (A), mean number of outcomes/drugs is presented (± standard deviation). In (B), number of patients is presented (which corresponds to percentages)

	MDD	Control	*P* value
A			
STOPP/START outcomes	3.65 ± 1.94	1.90 ± 1.64	<0.0001
STOPP outcomes	2.08 ± 1.48	0.97 ± 1.19	<0.0001
START outcomes	1.57 ± 1.34	0.93 ± 1.01	0.0004
Inappropriate/missing drugs (clinically relevant STOPP/START outcomes)	1.92 ± 1.52	1.06 ± 1.29	<0.0001
Inappropriate drugs (clinically relevant STOPP outcomes)	1.47 ± 0.70	0.70 ± 1.09	<0.0001
Missing drugs (clinically relevant START outcomes)	0.45 ± 0.64	0.36 ± 0.64	0.20
B			
≥1 STOPP/START outcome	97	73	<0.0001
≥1 STOPP outcome	86	54	<0.0001
≥1 START outcome	77	58	0.004
Suboptimal drug treatment (≥1 clinically relevant STOPP/START outcome)	86	55	<0.0001
Overtreatment (≥1 clinically relevant STOPP outcome)	75	43	<0.0001
Undertreatment (≥1 clinically relevant START outcome)	37	28	0.17

*MDD* multidose drug dispensing, *START*, Screening Tool to Alert to Right Treatment, *STOPP* Screening Tool of Older Person’s potentially inappropriate Prescription

In MDD and control patients, 147 out of 208 (70.7 %) and 70 out of 97 (72.2 %) STOPP outcomes were assessed as clinically relevant. For START outcomes, the corresponding figures were 45 out of 157 (28.7 %) and 36 out of 93 (38.7 %), respectively. MDD patients had an additional 0.77 inappropriate drugs and 0.09 missing drugs. Suboptimal drug treatment was more frequent in MDD patients than in control patients (86 vs. 55 %, *P* < 0.0001). The corresponding figures for over- and undertreatment were 75 vs. 43 % (*P* < 0.0001) and 37 vs. 28 % (*P* = 0.17), respectively.

The odds for suboptimal drug treatment were 8.0 times greater in MDD patients after adjustments for age, sex, number of drugs, cognition, and residence (95 % confidence interval (CI) 2.4; 26.9). The corresponding odds for over- and undertreatment were 2.9 (1.1; 7.4) and 1.8 (0.8; 4.3), respectively.

In Table 3, suboptimal drug treatment is described. Common overtreatment included (i) benzodiazepines, neuroleptics, and long-term opiates in those prone to falls, (ii) aspirin without an apparent indication, and (iii) loop-diuretics without clinical signs of heart failure. Common undertreatment included history of cardiovascular disease without recommended secondary prevention.

**Table 3:** Suboptimal drug treatment, including inappropriate and missing drugs, identified in ≥5 patients. Values are presented as number of patients (which corresponds to percentages)

		MDD, *n* = 100	Control, *n* = 100
Benzodiazepines in those prone to falls	I	32	15
Aspirin at dose >150 mg day	I	16	7
Loop diuretic for dependent ankle oedema only, i.e. no clinical signs of heart failure	I	15	5
Aspirin with no history of coronary, cerebral or peripheral arterial symptoms, or occlusive arterial event	I	6	7
Long-term long-acting benzodiazepines	I	6	7
Neuroleptic drugs in those prone to falls	I	10	1
Beta-blocker with chronic stable angina	M	6	4
Vasodilator drugs known to cause hypotension in those with persistent postural hypotension	I	5	4
Long-term opiates in those with recurrent falls	I	8	1
Aspirin or clopidogrel with a documented history of atherosclerotic coronary, cerebral, or peripheral vascular disease in patients with sinus rhythm	M	6	3
Statin therapy with a documented history of coronary, cerebral, or peripheral vascular disease, where the patient’s functional status remains independent for activities of daily living and life expectancy is >5 years	M	3	6
Prolonged use of first generation antihistamines	I	6	1
Warfarin in the presence of chronic atrial fibrillation	M	5	2
Oestrogens without progestogen in patients with intact uterus	I	4	2
First generation antihistamines in those prone to falls	I	5	0
Duplicate drug classes	I	4	1
ACE inhibitor with chronic heart failure	M	4	1
Bisphosphonates in patients taking maintenance oral corticosteroid therapy	M	3	2

*ACE* angiotensin converting enzyme, *I* inappropriate drug, *M* missing drug, *MDD* multidose drug dispensing, *START* Screening Tool to Alert to Right Treatment, *STOPP* Screening Tool of Older Person’s potentially inappropriate Prescription

## Discussion

In the present study, we show that suboptimal drug treatment, that is, over- and/or undertreatment, is more common in MDD patients compared with patients who receive their drugs via ordinary prescriptions. Indeed, MDD patients had an additional 0.77 inappropriate drugs and an additional 0.09 drugs that were missing but considered important. Further, after differences in characteristics between the patient groups had been considered, MDD patients ran an eight-fold increased risk for suboptimal drug treatment, a three-fold increased risk for overtreatment, and a two-fold increased risk for undertreatment. Thus, our findings suggest that MDD is a prominent determinant for suboptimal drug treatment. This finding is particularly interesting since MDD has been introduced to facilitate and increase safety in drug handling for the patient and the health care. Indeed, new technology which aims to solve a problem may introduce new problems [13].

In the present study, overtreatment was more prevalent than undertreatment. This may not be surprising since MDD patients are treated with numerous drugs [6, 8], many of which are psychotropics, a drug class which is considered hard to withdraw [14]. However, the proportion of STOPP outcomes being clinically relevant was quite similar between MDD and control patients indicating that the results cannot be explained by differences in suitability of the criteria between patients with and without MDD.

Interestingly, undertreatment was more common in MDD patients although these patients had an additional 2.5 drugs per patient compared with control patients. However, the lower confidence limit passed the line of unity. The proportion of START outcomes being clinically relevant was greater in control than in MDD patients. This may be explained by the higher age of MDD patients. Indeed, initiation of preventive treatment can be questioned with a short expected survival time.

Some previous studies indicate that an MDD system including medication records may provide a better overview of the patients’ medication lists and reduce medication errors [15–17]. In addition, the initiation of MDD may be preceded by a medication review in order to improve drug treatment quality [18]. In the Swedish MDD system, medication records are provided as well as computerized checks for drug interactions [19]. Thus, our findings that MDD is associated with suboptimal drug treatment may be surprising, and underlying mechanisms need to be speculated upon.

First, MDD implies that all dispensable tablets which should be ingested concomitantly are delivered in machine-dispensed unit bags. This procedure may reduce patients’ knowledge of drugs [4], and consequently their attentiveness to effects and adverse reactions for specific drugs. The same may apply to care-takers and relatives. Indeed, nurses have been reported to lose knowledge regarding patients’ medications upon introduction of an MDD system [16]. This may make nurses less involved in drug treatment and less capable of picking up drug-related symptoms. Taken together, a reduced level of knowledge in key persons may have an impact on the quality of drug treatment.

Second, the medication record used for drugs prescribed within an MDD system may differ from that of drugs prescribed the usual way in healthcare. In such systems, the prescriber has to document drug treatment in both systems [20] and may experience an increased workload [16, 17]. This may affect prescribing practices and reduce reconsideration of drug treatment, and consequently have an effect on treatment quality. Further, such systems may increase the risk of medication errors [19–21].

Third, another explanation for suboptimal drug treatment in MDD patients is that the conditions of these patients may be more multifaceted and thus involve more treatment difficulties, illustrated by the variety of clinically relevant STOPP and START outcomes identified. Indeed, the severity and the multiplicity of diseases may have an impact on the prevalence of over- and undertreatment.

### Strengths and weaknesses

The most important strength of the present study is that we have made individual considerations when evaluating the quality of drug treatment. Indeed, previous studies have used general indicators to distinguish between appropriate and suboptimal treatment, and these may not be applicable at the individual level. Further, with our approach, a major concern of previous studies, confounding by indication, is diminished. In fact, when evaluating inappropriate and missing drug treatment in register-based studies, differences between patients with and without MDD may be hard to control for.

Another strength is that suboptimal drug treatment included both over- and undertreatment. This approach gives a more comprehensive picture of the quality of the drug treatment as compared to previous studies which focus on overtreatment [6, 8]. The inter-rater agreement was moderate, indicating that some subjectivity is involved in the assessments of STOPP and START. This is not surprising since a clinical assessment is, at least partly, a matter of opinion. Nevertheless, consensus could be reached in all cases.

The fact that we have analyzed hip fracture patients implies both strengths and weaknesses. Indeed, these patients may represent a relevant subgroup of older patients since hip fracture is a common and serious diagnosis in Sweden, where every fourth middle-aged woman will sustain a hip fracture during her lifetime, one out of three hip fracture patients is a man, and the mortality rate within 6 months after the fracture is 20 % [22]. Further, these patients are treated with many medications [23] and many of them use MDD [7]. In fact, evaluating drug treatment quality in hip fracture patients may make the differences between MDD and control patients smaller than could be expected in the general population, where patients without MDD can be expected to be healthier. Nevertheless, the prevalence of over- and undertreatment, especially those involving fall risk, may differ from those found in a general population of older people.

A limitation of the present study is the cross-sectional study design, which does not allow conclusions concerning causality between MDD and suboptimal drug treatment. Thus, we cannot rule out if MDD leads to suboptimal drug treatment, or if such drug treatment leads to MDD. However, a previous longitudinal analysis of drug treatment before and after transition to an MDD system may suggest that the former alternative is more probable [8]. Another limitation is that the assessors were not blinded to the fact as to whether the patient had MDD or not. This was not practically possible, since medical records had to be scrutinized. The process of independent assessments and consensus, however, may diminish this problem. Further, although extensive and comprising in all 87 criteria per patient, STOPP and START may not capture all forms of over- and undertreatment. Thus, we cannot exclude other forms of suboptimal drug treatment. Finally, the generalizability of the results may be an issue as MDD systems may differ between countries and continents. However, although the process of prescribing may vary, the delivery of tablets in unit bags is a feature in common.

## Conclusion

We found that patients with MDD had suboptimal drug treatment to a greater extent than patients who received their drugs via ordinary prescriptions. Thus, this study confirms that MDD is associated with safety concerns when it comes to quality of drug treatment. The results should be of interest for health care decision-makers and prescribers in countries that already have, or plan to introduce, dose dispensing systems and to people who design the prescribing properties within such systems.
